# Preparation of Various Nanomaterials via Controlled Gelation of a Hydrophilic Polymer Bearing Metal-Coordination Units with Metal Ions

**DOI:** 10.3390/gels8070435

**Published:** 2022-07-11

**Authors:** Daisuke Nagai, Naoki Isobe, Tatsushi Inoue, Shusuke Okamoto, Yasuyuki Maki, Takeshi Yamanobe

**Affiliations:** 1 School of Food and Nutritional Science, University of Shizuoka, 52-1 Yada, Shizuoka 422-8526, Shizuoka, Japan; isobe_45@icloud.com (N.I.); sokamoto@u-shizuoka-ken.ac.jp (S.O.); 2 Division of Molecular Science, Faculty of Science and Technology, Gunma University, 1-5-1 Tenjin-cho, Kiryu 376-8515, Gunma, Japan; t15301013@gunma-u.ac.jp (T.I.); yamanobe@gunma-u.ac.jp (T.Y.); 3 Department of Chemistry, Graduate School of Science, Kyusyu University, 744 Motooka, Fukuoka 819-0395, Fukuoka, Japan; maki@chem.kyushu-univ.jp

**Keywords:** gelation, polymer, palladium, gold, coordination, nanosheet, nanofiber

## Abstract

We investigated the gelation of a hydrophilic polymer with metal-coordination units (HPMC) and metal ions (Pd^II^ or Au^III^). Gelation proceeded by addition of an HPMC solution in *N*-methyl-2-pyrrolidone (NMP) to a metal ion aqueous solution. An increase in the composition ratio of the metal-coordination units from 10 mol% to 34 mol% (HPMC-34) increased the cross-linking rate with Au^III^. Cross-linking immediately occurred after dropwise addition of an HPMC-34 solution to the Au^III^ solution, generating the separation between the phases of HPMC-34 and Au^III^. The cross-linking of Au^III^ proceeded from the surface to the inside of the HPMC-34 droplets, affording spherical gels. In contrast, a decrease in the ratio of metal-coordination units from 10 mol% to 4 mol% (HPMC-4) decreased the Pd^II^ cross-linking rate. The cross-linking occurred gradually and the gels extended to the bottom of the vessel, forming fibrous gels. On the basis of the mechanism for the formation of gels with different morphologies, the gelation of HPMC-34 and Au^III^ provided nanosheets via gelation at the interface between the Au^III^ solution and the HPMC-34 solution. The gelation of HPMC-4 and Pd^II^ afforded nanofibers by a facile method, i.e., dropwise addition of the HPMC-4 solution to the Pd^II^ solution. These results demonstrated that changing the composition ratio of the metal-coordination units in HPMC can control the gelation behavior, resulting in different types of nanomaterials.

## 1. Introduction

Organic–inorganic hybrid materials, which consist of organic polymers containing inorganic metals dispersed at the nanometer scale, have generated a great deal of interest due to their unique properties such as flexibility, high transparency, high reactivity, and mechanical and thermal stabilities [1,2,3,4,5]. For example, nanofibers containing metal ions or nanoparticles are promising candidates for various applications including tissue engineering, blood vessels, drug delivery, protective clothing, filtration, catalysis, and sensors [6,7,8,9,10]. The most common method of fabricating such nanofiber is an electrospinning method using a polymer solution containing metal ions or nanoparticles. However, electrospinning requires expensive instruments, cumbersome operations, and high voltage, and thus it runs the risk of electrical shock. Dispersing metal nanoparticles in the polymer solution is also difficult [11,12,13]. Therefore, the development of a facile method for fabricating nanofibers is greatly desired. Nanosheet materials also have unique physical and chemical properties, which are derived from their two-dimensional nature [14,15,16,17,18]. Nanosheets can be synthesized with a bottom-up method [19,20,21,22,23], which occurs at the interface between an organic polydentate ligand and an aqueous layer containing metal ions. This approach has advantages compared to the top-down approach, which produces nanosheets by exfoliation of bulk layered materials such as graphene. First, the composition, structure, and other properties can be adjusted by selection of the ligand molecules and metal ions. Second, the produced nanosheets are not limited to layers of bulk materials. Therefore, a bottom-up synthesis broadens the diversity and utility of nanosheets.

**Figure 1:**
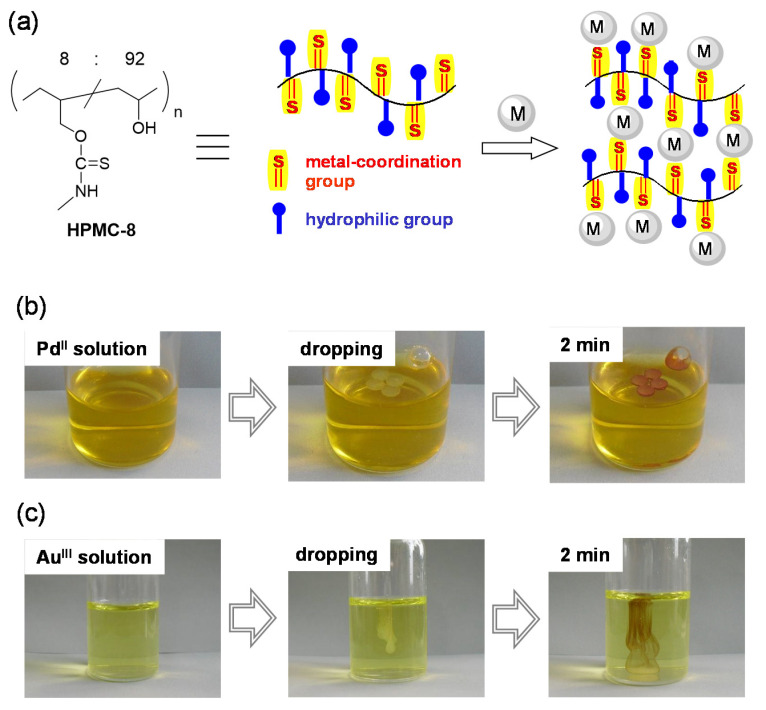
(**a**) Mechanism for gelation of HPMC―8 with metal ions. (**b**) Gelation behavior of HPMC―8 and Pd^II^ ions. (**c**) Gelation behavior of HPMC―8 and Au^III^ ions.

We previously investigated the gelation behavior of a hydrophilic polymer bearing metal-coordination units (denoted as HPMC) with metal ions (Pd^II^ or Au^III^) upon addition of a dispersed aqueous solution of HPMC-8 to an aqueous solution of metal ions [24,25]. HPMC-8 consists of thiocarbonyl groups (8 mol%) for metal coordination and hydroxyl groups (92%) for hydrophilicity (Figure 1a). The gelation of HPMC-8 with Pd^II^ or Au^III^ afforded spherical and fibrous gels, respectively (Figure 1b,c). Consequently, gels with different morphologies were found to be formed depending on the metal ions. The formation of different morphologies can be explained by the cross-linking rate. The cross-linking with Pd^II^ occurred immediately after dropwise addition of the dispersed aqueous solution of HPMC-8 to the Pd^II^ solution, generating the separation between aqueous phases of HPMC-8 and Pd^II^ (Figure 1b). The cross-linking of Pd^II^ proceeded from the surface to the inside of the droplets of HPMC, resulting in the formation of spherical gels. In contrast, the cross-linking with Au^III^ occurred gradually and the gels extended to the bottom due to the slower cross-linking rate, forming fibrous gels (Figure 1c). On the basis of this mechanism for the formation of gels with different morphologies, the gelation of HPMC-8 with Au^III^ provided nanofiber containing uniformly dispersed Au nanoparticles by a facile method, i.e., dropwise addition of a dispersed aqueous solution of HPMC-8 to an aqueous solution of Au^III^ ions [24]. In contrast, the faster gelation of HPMC-8 with Pd^II^ provided nanosheets containing uniformly dispersed Pd^II^ ions via gelation at the interface between the aqueous phases of Pd^II^ and HPMC-8 [25].

Encouraged by these results, we attempted to control the gelation behavior and synthesize different types of nanomaterials by changing the composition ratio of the metal-coordination units in HPMC. An increase in the composition ratio increased the cross-linking rate with Au^III^, resulting in the formation of spherical gels and Au nanosheets instead of nanofibers. A decrease in the ratio decreased the cross-linking rate with Pd^II^, affording fibrous gels and Pd nanofiber instead of nanosheets. Thus, changing the composition ratio of the metal-coordination units can provide contrasting gelation behavior and nanomaterials. We expect that this procedure will become a controlled manufacturing method for various types of nanomaterials containing various metals.

## 2. Results and Discussion

### 2.1. Synthesis of HPMC and Its Gelation Behavior with Metal Ions

**Scheme 1:**
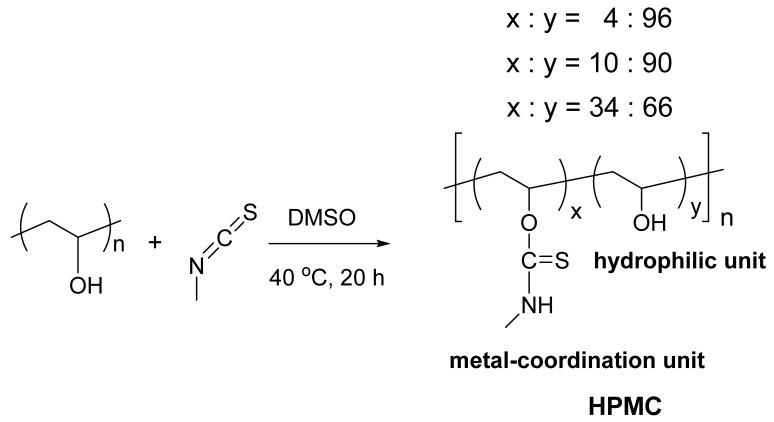
Synthesis of HPMCs by reactions of poly(vinyl alcohol) with methyl isothiocyanate.

HPMC containing thiocarbonyl and hydroxyl groups was synthesized according to our previous report [24]. HPMC with metal-coordination unit content of 10% (denoted as HPMC-10) was synthesized by reacting poly(vinyl alcohol) and methyl isothiocyanate in dimethyl sulfoxide at 40 °C for 20 h (Scheme 1). HPMC with metal-coordination unit content of 34% (HPMC-34) and HPMC with metal-coordination unit content of 4% (HPMC-4) were synthesized by the above similar method.

**Figure 2:**
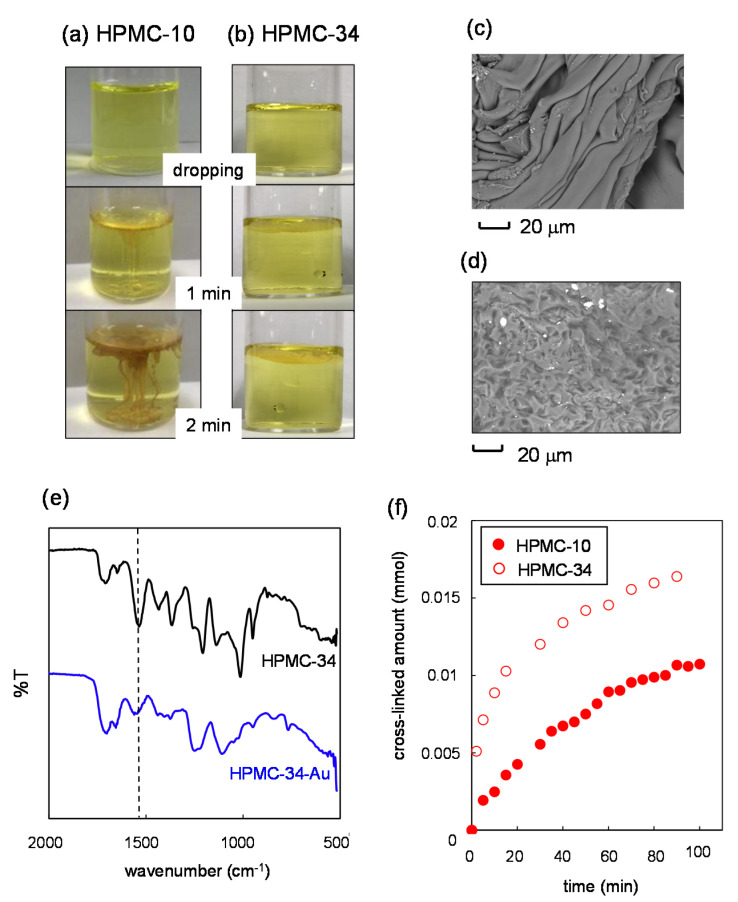
Gelation behavior of Au^III^ with (**a**) HPMC―10 and (**b**) HPMC―34. SEM images of (**c**) HPMC―10―Au and (**d**) HPMC―34―Au. (**e**) IR spectra of HPMC―34 and HPMC―34―Au. (**f**) Cross-linking rates of HPMC―10 and HPMC―34.

The gelation behavior of HPMC-34 and Au^III^ was compared to that of HPMC-10 and Au^III^ to examine the effect of the increase in the composition ratio of the metal-coordination unit. *N*-Methyl-2-pyrrolidone (NMP) solutions of HPMC-10 or HPMC-34 (13 wt%, 0.2 mL) were added to 4.0 mM NaAuCl_4_ aqueous solutions (5 mL). In the gelation of HPMC-10, Au^III^ ions gradually cross-linked from the surface to the inside phase of HPMC-10 and the gels extended to the bottom of the container, forming fibrous gels (Figure 2a). In contrast, gelation of HPMC-34 with Au^III^ generated instant separation between the phases of HPMC-34 and Au^III^ (Figure 2b). The separation originated from the immediate cross-linking reaction at the interface and the higher hydrophobicity of the HPMC-34 phase than that of the Au^III^ phase. The cross-linking with Au^III^ ions proceeded from the surface to the inside of the HPMC-34 droplets, resulting in the formation of spherical gels. To observe a microscopic region of the resulting gels, scanning electron microscope (SEM) observations were conducted. Similar to the gel shapes in the photographs (Figure 2a,b), SEM analysis showed fibrous shapes from HPMC-10-Au and a rough surface from HPMC-34-Au (Figure 2c,d). To determine the coordination sites, the IR measurements of HPMC-34 and HPMC-34-Au were carried out (Figure 2e). The absorption peak around 1535 cm^−^^1^ assigned to the C=S stretching vibration shifted to 1555 cm^−^^1^, and the peak intensity became smaller after the gelation. Therefore, it was found that different gelation behaviors and gel shapes were obtained depending on the composition ratio of the metal-coordination units.

A kinetic study and the gel fraction were examined to explain the mechanism for the formation of the different shaped gels. Cross-linking rates of HPMC-34 and HPMC-10 with Au^III^ ions were compared. Figure 2f shows the time course of the cross-linking amount determined by the method in the literature [24]. As shown in Figure 2f, the cross-linking rate of HPMC-34 was faster than that of HPMC-10 due to the increase in the metal-coordination units. The experimental kinetic data were fitted with a pseudo-first-order kinetic equation [26,27]:

log(*q*_e_ − *q*_t_) = *k*t/2.303 (1)

where *q*_e_ and *q*_t_ are the amounts of metal ion cross-linked (g_metal_/g_poly_, metal amount adsorbed per gram of polymer) at equilibrium and at t, and *k* is the pseudo-first-order rate constant (min^−1^). In the case of cross-linking reaction of HPMC-34, *k* was estimated to be 10.8 × 10^−2^ min^−1^ (*R*^2^ = 0.9712), which was faster than that for HPMC-10 (7.02 × 10^−2^ min^−1^, *R*^2^ = 0.9821). The gel fraction indicates the cross-linking density of the gels determined by removing soluble parts using Soxhlet extraction. Gel fractions of the gels from HPMC-34 and HPMC-10 were 0.76 and 0.14, respectively, indicating that the cross-linking density of HPMC-34-Au was higher than that of HPMC-10-Au.

On the basis of the results, a mechanism for the formation of the different morphologies was proposed. The cross-linking rate of HPMC-34 and Au^III^ was faster than that of HPMC-10. In the cross-linking of HPMC-34, gelation with Au^III^ occurred immediately at the surface of the droplets after the dropwise addition of the HPMC solution to the Au^III^ solution. Gelation proceeded by immersing Au^III^ inside of the droplets, forming the higher cross-linking density and spherical gels. Contrastingly, due to the slower cross-linking rate of HPMC-10, the cross-linking occurred gradually with the diffusion of Au^III^ from the surface to the inside of the HPMC-10 phase, resulting in the formation of the lower cross-linking density and fibrous gels.

**Figure 3:**
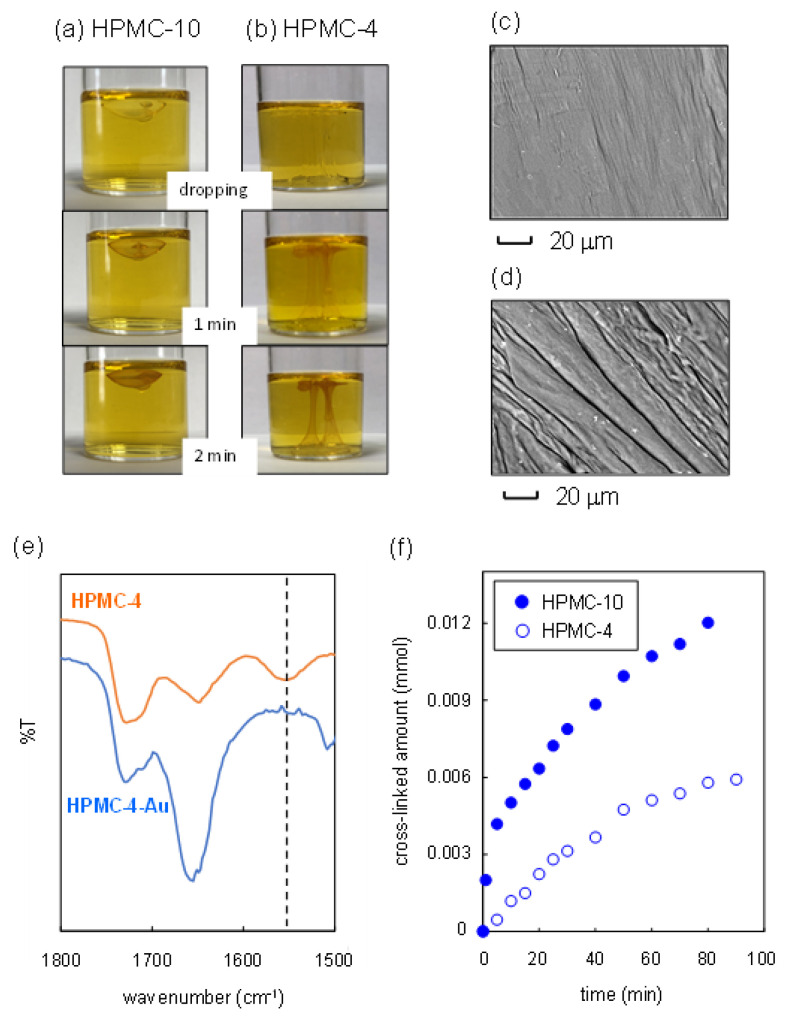
Gelation behavior of Pd^II^ with (**a**) HPMC―10 and (**b**) HPMC―4. SEM images of (**c**) HPMC―10―Pd and (**d**) HPMC―4―Pd. (**e**) IR spectra of HPMC―4 and HPMC―4―Au. (**f**) Cross-linking rates of HPMC―4 and HPMC―10.

Next, to examine the effect of the decrease in the ratio of metal-coordination units on the gelation, the gelation behavior of HPMC-4 and Pd^II^ was compared to that of HPMC-10 with Pd^II^. NMP solutions of HPMC-10 or HPMC-4 (13 wt%, 0.2 mL) were added to 4.0 mM Na_2_PdCl_4_ aqueous solutions (5 mL). In the gelation of HPMC-10, immediate separation occurred between the HPMC-10 and Pd^II^ phases (Figure 3a), whose separation originated from the fast cross-linking at the interface. The cross-linking with Pd^II^ proceeded from the surface to the inside of the HPMC-10 droplets, affording the spherical gels. In contrast, gelation of HPMC-4 and Pd^II^ occurred gradually from the surface to the inside of HPMC-4 phase, and the gels were extended to the bottom of the container, forming the fibrous gels. To observe a microscopic region, SEM analysis of the obtained gels was conducted. Similar to the gel shapes (Figure 3a,b), the SEM analysis revealed a rough surface from HPMC-10-Pd and fibrous shapes from HPMC-4-Pd (Figure 3c,d). To determine the coordination site, IR measurements of HPMC-4 and HPMC-4-Pd were conducted (Figure 3e). The absorption peak at 1550 cm^−1^ attributable to the C=S stretching vibration became smaller after cross-linking, indicating that the sulfur of the thiocarbonyl group was coordinated to Pd^II^.

As shown in Figure 3f, the cross-linking rate of HPMC-4 was slower than that of HPMC-10 due to the decrease in the metal-coordination units. The pseudo-first-order kinetic rate constants, *k*s of HPMC-4 and HPMC-10 were estimated to be 6.38 × 10^−2^ min^−1^ (*R*^2^ = 0.9821) and 8.62 × 10^−2^ min^−1^ (*R*^2^ = 0.9712), respectively. The gel fractions of the gels from HPMC-4 and HPMC-10 were 0.01 and 0.17, respectively, indicating that the cross-linking density of HPMC-4 was lower than that of HPMC-10. Consequently, the cross-linking reaction of HPMC-4 gradually proceeded with the diffusion of Pd^II^ from the surface to the inside of the HPMC phase due to the slower cross-linking rate, providing lower cross-linking density and fibrous gels as opposed to the gelation of HPMC-10 (spherical gels).

### 2.2. Synthesis of Nanosheets

As described above, the dropwise addition of the NMP solution of HPMC-34 to the Au^III^ aqueous solution allowed instant separation of the HPMC-34 and Au^III^ phases (Figure 2b). The liquid/liquid separation originated from the fast cross-linking at the interface and the higher hydrophobicity of the HPMC-34 phase than the Au^III^ phase. The Au^III^ ions cross-linked from the surface to the inside of the HPMC-34 droplets, affording spherical gels. This feature prompted us to utilize the cross-linking at the liquid–liquid interface between the HPMC-34 and Au^III^ phases for the synthesis of nanosheets.

**Figure 4:**
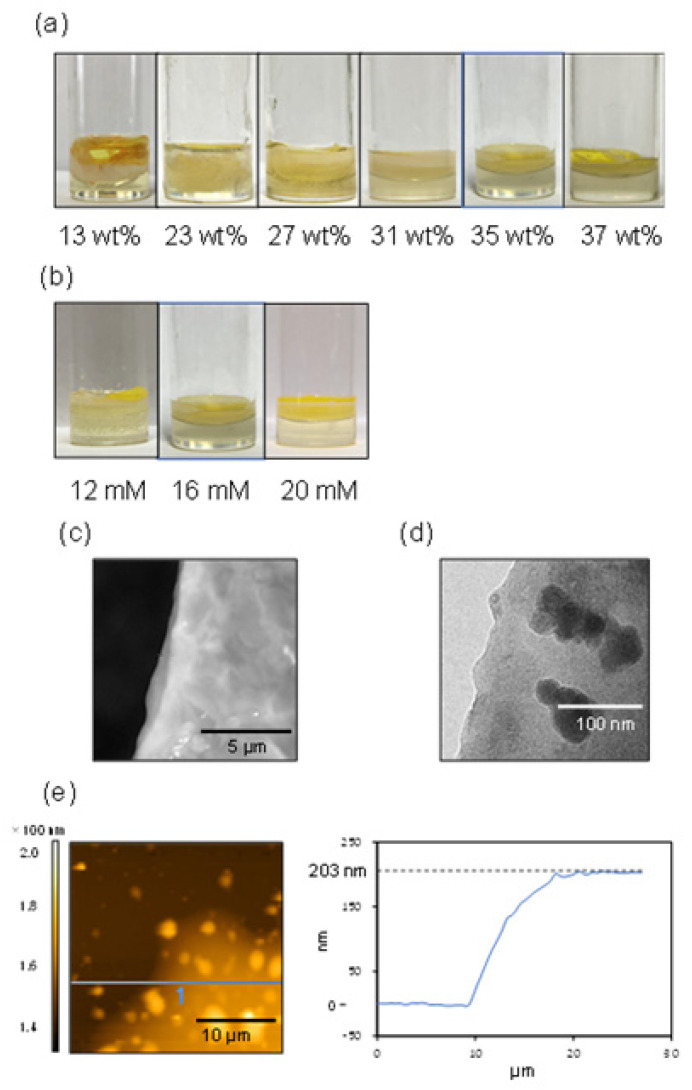
Photographs of (**a**) bottom-up synthesis of nanosheets by the addition of Au^III^ aqueous solutions (16 mM) to NMP solutions of HPMC―34 (13―37 wt%) and (**b**) bottom-up synthesis of nanosheets by the addition of Au^III^ aqueous solutions (12―20 mM) to NMP solutions of HPMC―34 (35 wt%). (**c**) SEM image of HPMC―34―Au nanosheet. (**d**) TEM image of HPMC―34―Au nanosheet. (**e**) AFM image of HPMC―34―Au nanosheet on a Si substrate.

The synthesis of nanosheets was attempted by the generation of the interface using solutions with different specific gravities and fast cross-linking between thiocarbonyl groups of HPMC-34 and Au^III^ ions (i.e., dropwise addition of an aqueous solution of Au^III^ ions with a lower specific gravity (1.02 g/cm^3^) to an NMP solution of HPMC-34 with a higher specific gravity (1.63 g/cm^3^)). When the Au^III^ aqueous solutions (16 mmol/L, 0.4 mL) were gently added to the NMP solutions of HPMC-34 with different concentrations (13, 23, and 27 wt%), the upper Au^III^ solution was miscible with the lower HPMC-34 concentration due to the slow cross-linking rate. In contrast, the increase in the concentration of HPMC-34 to 31, 35, and 37 wt% allowed instant cross-linking leading to the liquid/liquid separation, resulting in the formation of film-shaped gels at the interface (Figure 4a). Next, to examine the effect of Au^III^ concentration, aqueous solutions of Au^III^ with different concentrations (12, 16, and 20 mmol) were added to the HPMC-34 solutions (35 wt%). In every case, liquid/liquid separations were observed (Figure 4b). The thin film that formed at the interface between the Au^III^ (20 mmol) and HPMC (35 wt%) phases was transferred onto a Petri dish using tweezers, followed by washing with NMP and drying under reduced pressure. SEM and transmission electron microscope (TEM) images revealed the formation of a sheet structure (Figure 4c,d). Atomic force microscopy (AFM) showed a thickness of approximately 203 nm (Figure 4e). These results demonstrate the successful formation of the nanosheets at the interface between the Au^III^ and HPMC phases. The obtained nanosheet was characterized structurally. As shown in Figure 2c, the coordination site of Au was through the thiocarbonyl groups. The XPS wide-scan spectrum showed a peak of Au 4f around 84.0 eV and no peak of Cl 2p around 200 eV (see Supplementary Materials Figure S1a). The XPS narrow-scan spectrum showed Au 4f_7/2_ and Au 4f_5/2_ peaks at 83.9 and 87.6 eV, respectively, which are typical of Au^0^ species [28,29,30]. (see Supplementary Materials Figure S1b). These results indicate that the Au^III^ was reduced to Au^0^ during gelation, similar to our previously proposed mechanism [24].

### 2.3. Synthesis of Nanofiber

**Figure 5:**
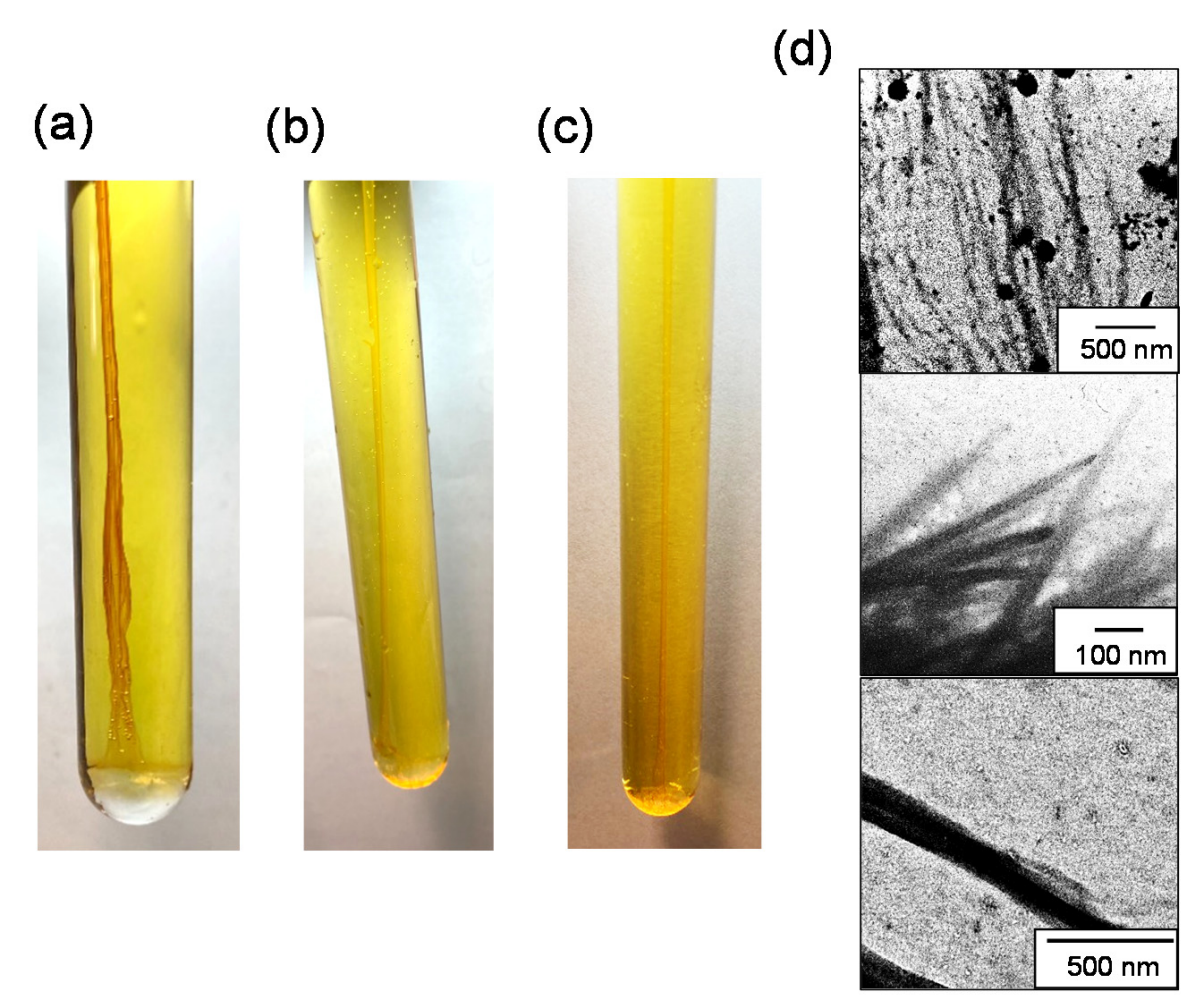
Photographs of gels produced upon addition of NMP solutions of (**a**) 11 wt%, (**b**) 8 wt%, and (**c**) 6 wt% of HPMC―4 (0.2 mL) to 4 mM aqueous solutions of Pd^II^ ions (20 mL). (**d**) TEM images of HPMC-4-Pd nanofiber.

As mentioned above, the gelation of HPMC-4 with Pd^II^ ions provided fibrous gels with a lower cross-linking rate and the stretching force to the bottom of the container induced by the gel weight. Nanofibers containing metal ions are generally synthesized by the electrospinning method using a polymer solution containing metals. However, electrospinning has various problems, as described in Section 1. Thus, the synthesis of nanofibers was attempted by a facile method, i.e., dropwise addition of an NMP solution of HPMC-4 to an aqueous solution of Pd^II^ ions. HPMC-4 solutions (6, 8, and 11 wt%) were added to a NaAuCl_4_ aqueous solution (4 mM) in a test tube. In all cases, elongated gels were obtained (Figure 5a–c). Elongation of gels increased with decreasing polymer concentration. The TEM observation of the gels obtained at 6 wt% of polymer concentration revealed the formation of fibrous gels of 100~200 nm diameter. The XPS narrow-scan spectrum of the nanofiber showed Pd 3d_5/2_ and Pd 3d_3/2_ peaks at 336.8 eV and 342.1 eV, respectively, which are typical of Pd^II^ species (Figure S2a) [28,31]. EDX/SEM measurement showed the presence of Pd and Cl species (Figure S2b). The Cl species was ascribed to Na_2_PdCl_4_; therefore, Pd^II^Cl_2_ was contained in the nanofibers. Thus, nanofibers cross-linked with Pd^II^ ions were successfully synthesized by this dropwise addition method.

## 3. Conclusions

In conclusion, we demonstrated the control of gelation behavior for the synthesis of different types of nanomaterials by changing the composition ratio of the metal-coordination unit in HPMC. An increase in the composition ratio of the metal-coordination unit from 10 mol% to 34 mol% increased the cross-linking rate with Au^III^, resulting in the formation of spherical gels and Au nanosheets. A decrease in the ratio of the metal-coordination unit from 10 mol% to 4 mol% decreased the cross-linking rate with Pd^II^, affording fibrous gels and Pd nanofibers. Changing the composition ratio of the metal-coordination unit allowed the contrasting gelation behavior to form various types of nanomaterials with metal ions. This procedure will enable the controlled synthesis of various types of nanomaterials containing various metals, which is now under investigation.

## 4. Materials and Methods

### 4.1. Materials

Polyvinyl alcohol (PVA, average polymerization degree = 1200) (Wako Pure Chemical,) was used as received. Methyl isothiocyanate (Tokyo Kasei Kogyo, >98.0%) was distilled prior to use. Sodium tetrachloropalladate (II) (Na_2_PdCl_4_, Tokyo Kasei Kogyo, >98.0%) and sodium tetrachloroaurate (III) dehydrate (NaAuCl_4_, Wako Pure Chemical, >95.0%) were commercially available and used as received. Dimethylsulfoxide (Wako Pure Chemical, >99.0%) was distilled under CaH_2_. N-Methylpyrrolidone (NMP, Wako Pure Chemical, >99.0%) was used as received.

### 4.2. Instruments

^1^H NMR spectra were measured with a JEOL JNM ECA-500 using tetramethylsilane (TMS) as an internal standard; δ values are given in parts per million (ppm). IR spectra were measured with a SHIMADZU FTIR IRPrestige-21 spectrometer, and the values are provided in cm^−^^1^. Flame atomic absorption spectrometry was conducted with a Hitachi Z-2310 polarized Zeeman atomic absorption spectrometer (AAS). X-ray photoelectron spectroscopy (XPS) was performed with a Kratos AXIS-NOVA instrument. Scanning electron microscopy (SEM) was performed using a HITACHI S-2500 instrument at an acceleration voltage of 1.5 kV. Energy-dispersive X-ray analysis (EDX/SEM) was conducted using a HITACHI S-3400N/BRUKER Quantax 200 System. Transmission electron microscopy (TEM) was conducted using a HITACHI HT-7700 instrument at an acceleration voltage of 20 kV. Samples for TEM analysis were deposited onto a Cu grid. Atomic force microscopy (AFM) was conducted with a KEYENCE VN-8010 instrument using a silicon substrate in the high amplitude mode (tapping mode) under an ambient condition.

### 4.3. Gelation of HPMC and Metal Ions

NMP solutions of HPMC (0.200 mL, 13 wt%) were added to 4.00 mM aqueous solutions of Na_2_PdCl_4_ (10.0 mL) or NaAuCl_4_ (10.0 mL). Gelation was conducted at room temperature for 2 min, and the obtained gel was dried to constant weight at 60 °C in vacuo.

The gel fraction of the cross-linked HPMC was determined gravimetrically. The dry gels were washed with refluxed distilled water in a Soxhlet extraction to remove the soluble parts. The washed gels were dried to constant weight at 80 °C in vacuo. The gel fraction was calculated from the following equation:

Gel fraction = *W*_wash__—dry_/*W*_dry_

where *W*_dry_ is the weight of the dried gel before Soxhlet extraction and *W*_wash__—dry_ is the weight of the dried gel after Soxhlet extraction.

### 4.4. Synthesis of Au Nanosheets

An aqueous solution of Au^III^ ions (20.0 mM, 0.800 mL) was carefully added to an NMP solution of HPMC-34 (31.0 wt%, 0.800 mL) in a vial bottle. After 3 s, the obtained nanosheets were transferred from the interface between the Au^III^ ion layer and HPMC-34 layer onto a Petri dish. The nanosheets were washed with NMP, followed by drying under vacuum.

### 4.5. Synthesis of Pd Nanofibers

An NMP solution of HPMC-4 (0.200 mL, 6.00 wt%) was added to a 25.0 mL test tube containing 4.00 mM Na_2_PdCl_4_ aqueous solution (20.0 mL), and the mixture was allowed to stand at room temperature for 4 min. The resulting gels were handled using tweezers and placed in a Petri dish. The gels were washed with NMP, followed by drying to obtain the fibrous gels.

## Supplementary Materials

The following supporting information can be downloaded at: https://www.mdpi.com/article/10.3390/gels8070435/s1, Figure S1: Stability of HPMC-10-Au; Figure S2: Stability of HPMC-34-Au; Figure S3: TEM images of HPMC-34-Au nanosheet; Figure S4: XPS spectra of Au^o^ nanosheet: (a) wide-scan spectrum and (b) narrow-scan spectrum; Figure S5: (a) EDX spectrum of PdII nanofiber. (b) XPS narrow-scan spectrum of PdII nanofiber.
