# Supplementary material for: Preparation of Various Nanomaterials via Controlled Gelation of a Hydrophilic Polymer Bearing Metal-Coordination Units with Metal Ions
Source: Gels. 2022 Jul 11;8(7):435. doi: 10.3390/gels8070435 (PMC9322127; doi:10.3390/gels8070435)
Supplement: Supplementary file 1 [file gels-08-00435-s001.zip › gels-1766400-supplementary.pdf]

Preparation of Various Nanomaterials via Controlled Gelation of a Hydrophilic Polymer Bearing Metal-Coordination Units with Metal Ions

Daisuke Nagai, Naoki Isobe, Tatsushi Inoue, Shusuke Okamoto, Yasuyuki Maki, and Takeshi Yamanobe*

Table of Contents

Figure S1	S2
Figure S2	S2
Figure S3	S2
Figure S4	S3
Figure S5	S4

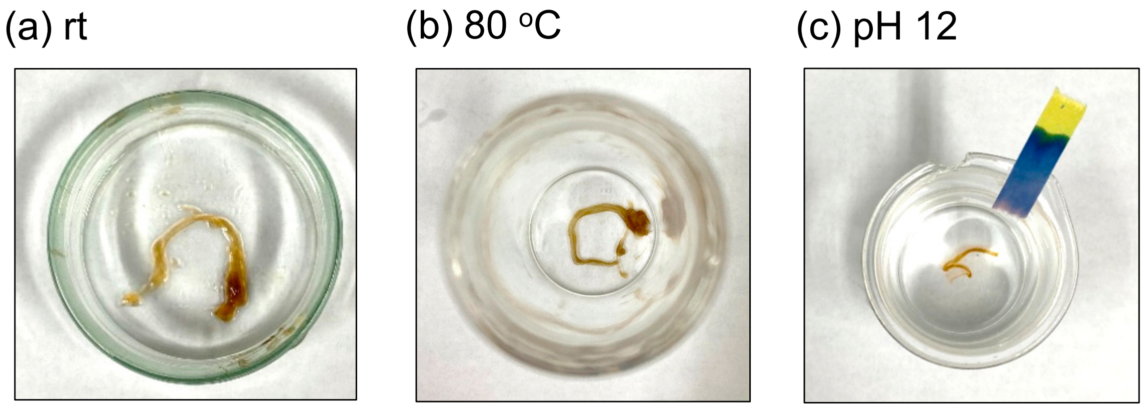

Figure S1. Stability of HPMC-10-Au

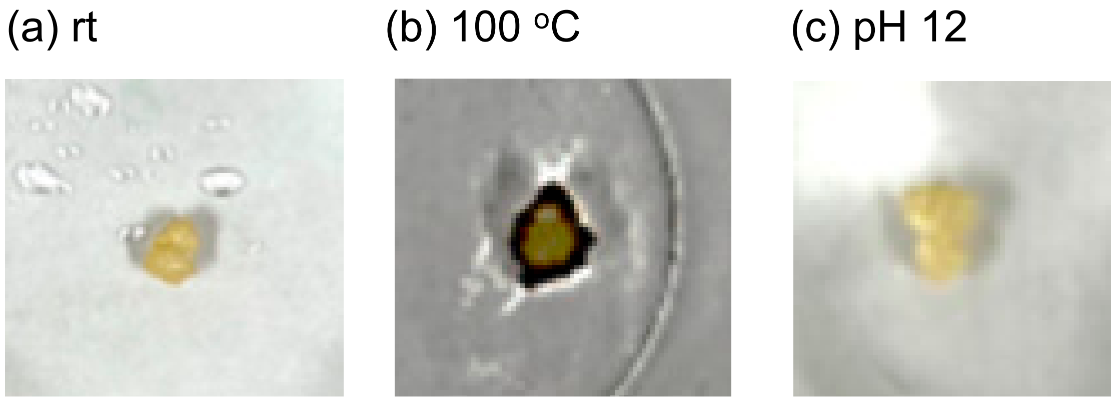

Figure S2. Stability of HPMC-34-Au

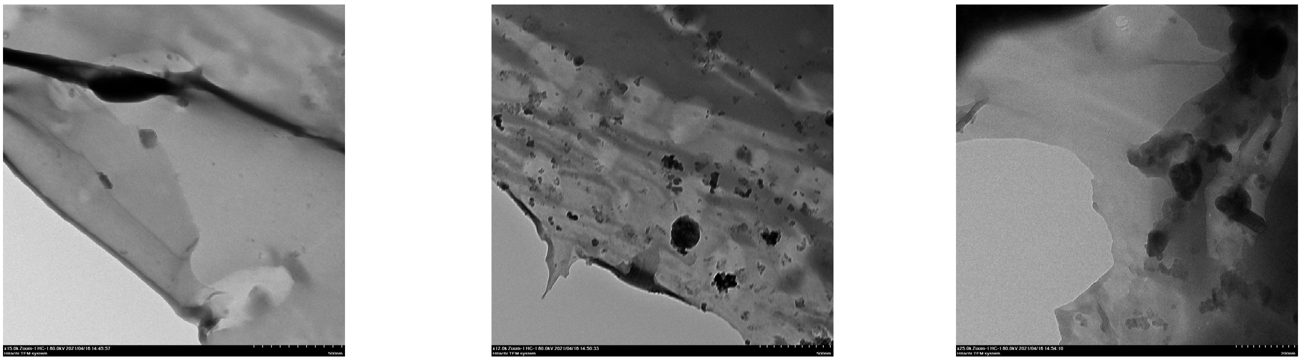

Figure S3. TEM images of HPMC-34-Au nanosheet.

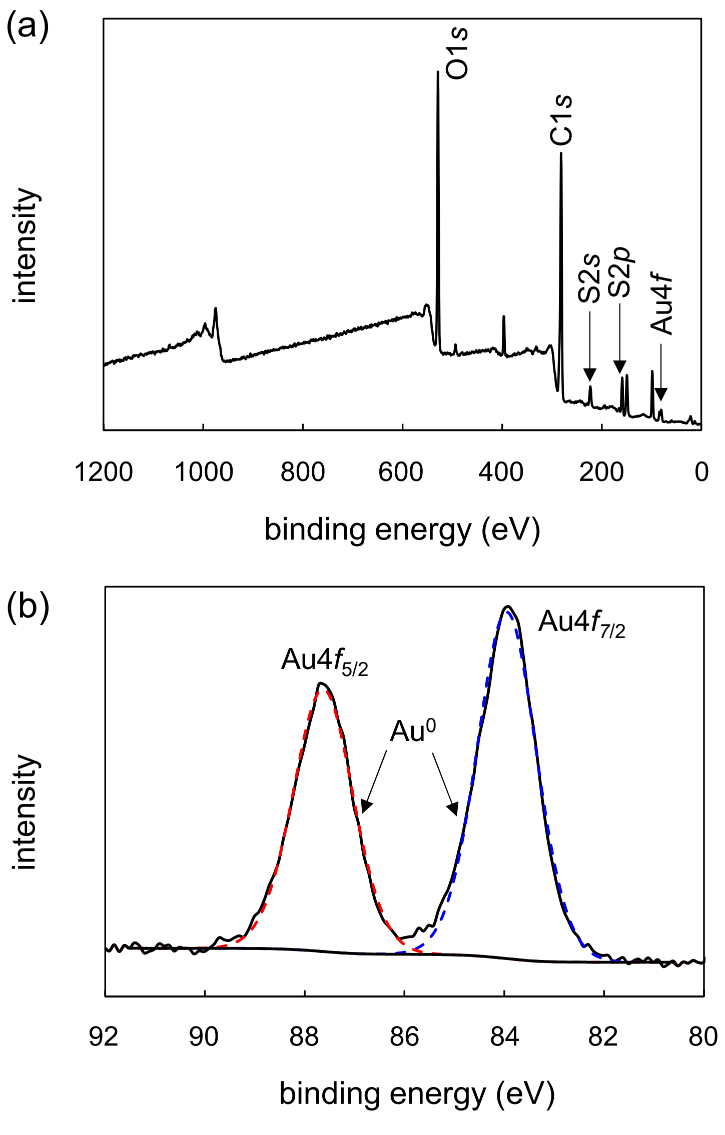

Figure S4. XPS spectra of Au^{o} nanosheet : (a) wide-scan spectrum and (b) narrow-scan spectrum.

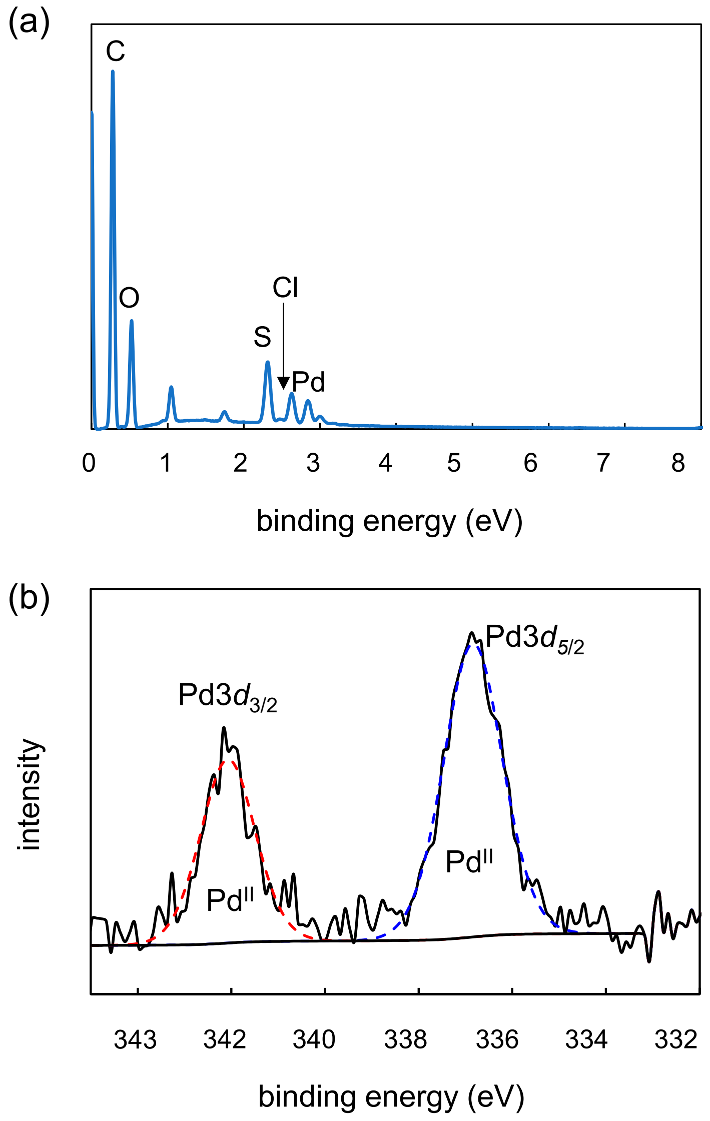

Figure S5. (a) EDX spectrum of Pd^{II} nanofiber. (b) XPS narrow-scan spectrum of Pd^{II} nanofiber.
